# Enhancing postural stability in a musculoskeletal hopping robot through stretch reflex application on biarticular thigh muscles

**DOI:** 10.3389/frobt.2023.1293365

**Published:** 2023-11-23

**Authors:** Ryu Takahashi, Yuki Murakami, Koh Hosoda

**Affiliations:** ^1^ Adaptive Robotics Laboratory, Graduate School of Engineering Science, Osaka University, Toyonaka, Japan; ^2^ Graduate School of Engineering, Kyoto University, Kyoto, Japan

**Keywords:** hopping, legged design, legged robot, postural stability, pneumatic artificial muscles, stretch reflex

## Abstract

Postural stabilization during rapid and powerful hopping actions represents a significant challenge for legged robotics. One strategy utilized by humans to negotiate this difficulty is the robust activation of biarticular thigh muscles. Guided by this physiological principle, this study aims to enhance the postural stability of a hopping robot through the emulation of this human mechanism. A legged robot powered by pneumatic artificial muscles (PAMs) was designed to mimic human anatomical structures. A critical aspect of this development was creating a tension-oriented stretch reflex system engineered to initiate muscle activation in response to perturbations. Our research encompassed three experiments: 1) assessing the trunk pitch angle with and without the integration of stretch reflexes, 2) evaluating the consistency of hops made with and without reflexes, and 3) understanding the correlation between the reflex strength equilibrium in the biarticular thigh muscles and trunk pitch angle. The results indicated that the integration of the stretch reflex minimized perturbations, thereby allowing the robot to perform double the continuous hops. As hypothesized, adjusting the reflex strength equilibrium caused a shift in the angle. This reflex mechanism offers potential application to PAM-driven robots and signifies a promising avenue for enhancing postural stability in diverse forms of locomotion, including walking and running.

## 1 Introduction

Hopping is a challenging task for legged robots, as it requires the generation of strong vertical forces coupled with dynamic postural stabilization, inclusive of collision management. Humans execute this task seamlessly, owing the complex morphological attributes of our musculoskeletal system (Pfeifer and Bongard, 2006; Neumann, 2016; Sharbafi and Seyfarth, 2017). Multiple investigations have been conducted to enhance robotic locomotion by replicating these attributes. One prominent example is legged robots powered by pneumatic artificial muscles (PAMs), which mirror human muscular characteristics regarding flexibility and linear actuation. Their antagonistic placement at joint locations provides elasticity and produces vertical forces for hopping (Dowling and Vamos, 1993; Kubo et al., 1999; Roberts and Azizi, 2011; Mohseni et al., 2021; Zhao et al., 2022). Moreover, the configuration across multiple joints offers redundancy in joint degrees of freedom, resulting in efficient force transmission at each joint (Gregoire et al., 1984; Pandy et al., 1990; Zajac et al., 2002; Ruppert and Badri-Spröwitz, 2019). These features allow PAM-driven robots to perform dynamic hopping (Daerden et al., 2001; Niiyama et al., 2007; Hosoda et al., 2008; Hosoda et al., 2010; Kaneko et al., 2016). However, in-depth studies on postural stabilization remain scarce due to the complex control over PAMs’ nonlinear dynamics, with no documented examples of such robots achieving continuous hopping in the sagittal plane.

Conventional robots implement postural stabilization via a rapid feedback mechanism, obtaining posture data from IMUs and encoders. Conversely, human postural control relies on neural systems and musculoskeletal system with extended latencies, suggesting an inherent self-stabilization system with minimal control input (Winter, 2009). This capability can be attributed to biarticular muscles that produce torque across multiple joints. In a human static stance perturbation test, the rectus femoris (RF) and hamstrings (HAM), biarticular thigh muscles, displayed higher activity compared to other leg muscles (Schumacher et al., 2019). When subjects maintained a unipedal stance while external forces acted upon the unsupported leg in both anterior and posterior directions, both RF and HAM exhibited consistent counteractive muscle activities. However, monoarticular muscle activities exhibited less regularity (Hosoda et al., 2017). Significant RF and HAM activity was not just confined to standing but also evident during ambulatory activities (Prilutsky et al., 1998), weight-lifting (Toussaint et al., 1992), and cycling (van Ingen Schenau et al., 1992). Template models suggest that these muscles can generate a Ground Reaction Force (GRF) for hip rotation, independent of knee joint angle, given a 2:1 ratio between moment arms to the hip and knee and equal lengths of the thigh and lower leg (Lakatos et al., 2014; Hosoda et al., 2017).

Humans employ various neural control strategies to initiate muscle response against postural perturbations (Johansson and Magnusson, 1991; Horak, 2006; Mergner, 2010; van de Ruit et al., 2018). The foremost immediate reaction mechanism is the spinal reflex, especially the stretch reflex, which induces rapid muscle contraction upon stretching through muscle spindles, thereby activating muscle responses within 40 ms (Voigt et al., 1998; Zuur et al., 2010). While the exact operative mechanism behind RF and HAM muscle activity remains elucidated, the stretch reflex emerges as a viable candidate. Several control models replicating stretch reflexes for robotic systems have been developed (Geyer et al., 2003; Geyer and Herr, 2010; Song and Geyer, 2015; Schumacher and Seyfarth, 2017; Kawaharazuka et al., 2020; Mohseni et al., 2022), and their potential benefits have been reported for PAM-driven robots as well (Rosendo et al., 2015; Liu et al., 2018).

This study implements a stretch reflex system on the biarticular thigh muscles to enhance the postural stability of a PAM-driven hopping robot. A robot equipped with 12 PAMs corresponding to the eight muscles required for hopping is developed. Subsequently, a tension-oriented stretch reflex system is integrated. The main objective is to evaluate the efficacy of this approach in reducing the trunk pitch perturbation during hopping trials with an actual robot. Here, three experimental procedures were executed: 1) measuring trunk pitch angle with and without stretch reflexes from the moment the free-fall robot landed to its subsequent rebound and landing, 2) investigating the number of continuous hops with and without stretch reflexes, and 3) exploring the correlation between the reflex strength equilibrium in the biarticular thigh muscles and trunk pitch angle.

## 2 Methods

Hopping has a hybrid dynamic comprising stance and flight phases, and its control comprises three controllers: stance, flight, and balance (Raibert, 1986; Sharbafi and Seyfarth, 2017). These functions collectively oversee vertical hopping control by pushing off the ground, mid-air landing posture adjustments, and postural controls to minimize body rotation. This study integrated the stretch reflex on the biarticular thigh muscles as a balance function and adopted controls similar to those in our previous studies for stance and flight (Hosoda et al., 2010). In this section, we describe the concept of the proposed stretch reflex system and its implementation methodology, as well as the stance and flight control. Finally, a technique for identifying the timing of transitions between each function is provided.

### 2.1 Tension-oriented stretch reflex for posture stability

A tension-oriented stretch reflex on the biarticular thigh muscle has been proposed as a novel balance function suitable for a PAM-driven legged robot. It has been reported that the muscles, specifically rectus femoris (RF) and hamstrings (HAM), play a pivotal role in human strategies to manage postural perturbations (Schumacher et al., 2019). Mathematical analysis using a template model aligns with this observation (Lakatos et al., 2014; Hosoda et al., 2017). These muscles can produce a GRF for hip rotation independent of the knee joint angle when the moment arms connected to the hip and knee joints maintain a 2:1 ratio and both the thigh and lower leg lengths are equivalent.

**FIGURE 1:**
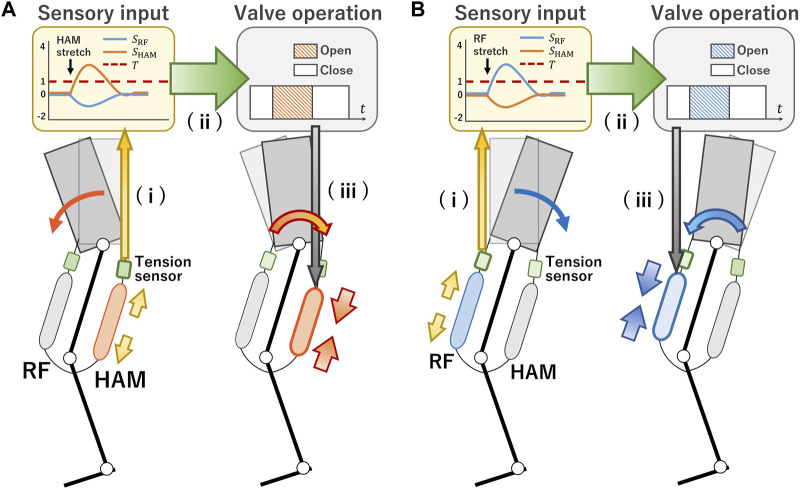
Concept of stretch reflex system. **(A)** (i) When the robot’s trunk leans forward, the HAM stretches significantly, increasing its tension while the tension in RF decreases. (ii) The system obtains the data from the tension sensors and produces a valve operation command, causing the muscle to contract and preventing further stretching of the HAM. (iii) The HAM’s contraction produces a hip torque that corrects the forward lean of the trunk. **(B)** Conversely, when the robot’s trunk leans backward, the opposite behavior occurs in the RF.

Our hypothesis postulated that postural variations could be discerned from sensory information derived from the biarticular thigh muscles. They can be activated through the stretch reflex to stabilize the posture. In the information process of the human stretch reflex, alterations in muscle length detected by muscle spindles are conveyed directly to motor neurons. These neurons are triggered to produce action potentials when the alterations surpass a certain threshold. Bearing technical feasibility in mind, we replicated this process employing a tension sensor. Figure 1 illustrates a schematic representation of the proposed approach. The robot is equipped with PAMs and tension sensors positioned antagonistically on the thigh. As hopping incurs postural perturbation, one of the PAMs undergoes stretching while its counterpart relaxes. This action produces a respective tension that the robot’s control system interprets and processes. For example, if the robot’s trunk leans forward, the HAM experiences extensive stretching, elevating its tension, while the tension in the RF decreases [refer to Figure 1A, (i)]. The system obtains the data from the tension sensors and prompts a valve operation command to initiate muscle contraction. This action prevents the HAM from excessive stretching [see Figure 1A, (ii)]. The contraction of the HAM results in a hip torque, realigning the forward-leaning trunk to its neutral stance [as depicted in Figure 1A, (iii)]. Conversely, when the robot’s trunk is reclined, the RF exhibits the opposite behavior, as highlighted in Figure 1B.

**FIGURE 2:**
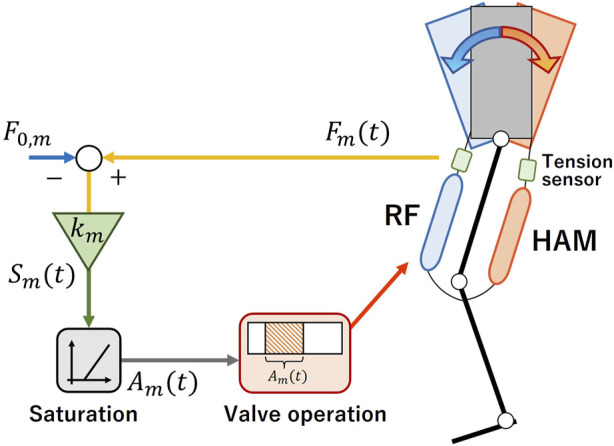
Schematic diagram of stretch reflex system.

The conversion process from sensory information to valve operation commands, as depicted in Figures 1A, B, (ii), is further detailed in Figure 2. The system captures the tension produced within the target muscle and, at each control cycle, computes the extent of muscle stimulation by amplifying the tension with the control gain. The air supply duration to the muscle is defined by this magnitude. This process can be mathematically represented as:

$$S_{\text{m}}\left(t\right)=k_{\text{m}}\left(F_{\text{m}}\left(t\right)-F_{0,m}\right)$$ (1)

$$A_{\text{m}}\left(t\right)=\left\{\begin{array}{cc} 0\; & \left(S_{\text{m}}\left(t\right)<\alpha \right)\\ S_{\text{m}}\left(t\right)T_{\text{m}}\; & \left(S_{\text{m}}\left(t\right)\geq \alpha \right) \end{array}\right.$$ (2)

where the subscript m refers to either the RF or HAM. *F*_m_ denotes the tension within muscle m, while *F*_0,m_ stands for the initial tension offset. Employing Eq. 1, the system calculates the magnitude of the muscle stimulation *S*_m_ from the tension values and the control gain *k*_m_. The PAM is directed by the solenoid valve’s opening and closing duration *A*_m_, which is calculated from the stimulation *S*_m_ and the reference duration *T*_m_ using Eq. 2. The rationale behind converting stimulation *S*_m_ to air supply duration *A*_m_ lies in the valve’s limited functionality—solely opening and closing. While a PWM signal could theoretically represent amplitude modulation against the tension sensor value, the valve’s opening and closing frequency falls short compared to a typical PWM signal. Thus, a method of converting sensor values to air supply duration was chosen to simulate the amplitude modulation of the input to the valves with a smaller frequency.

Here, *α* symbolizes the threshold, mimicking the stimulation intensity that triggers an action potential. This sequence prompts the PAM to contract upon receiving stimulation. When the value of *S*_m_ surpasses the threshold *α*, the duration of *A*_m_ is initiated. Though *S*_m_ receives regular updates at a 250 Hz, the sampling rate of the tension sensor, it doesn’t influence the valve operation until the course of *A*_m_ is complete. The computer’s control frequency, which sends commands to the valve, stands at 1 kHz and ensure timely response to updates in *A*_m_. These frequencies are determined by the hardware constraint, yet they align closely with the characteristics of human neural responses. In the experiment, *T*_m_ was consistently set at 5 ms (Table 2). Although there is no upper limit to the dimensionless value of *S*_m_, it typically falls within the range of 0–6 based on the tension sensor value and magnitude of the gain. Since the threshold value of *S*_m_ at which *A*_m_ is activated is 1, *S*_m_ *T*_m_ is expected to fall within the range of 5–30 ms. The intrinsic human stretch reflex mechanism can modulate the intensity of the reflex response based on the given task. Similarly, the robotic system can also adapt the intensity by altering the gain *k*_m_. The appropriate gains were set empirically to ensure that the duration of a single air supply (*A*_m_) would be reasonable for a stance period of approximately 250 ms.

### 2.2 Stance and flight function

**FIGURE 3:**
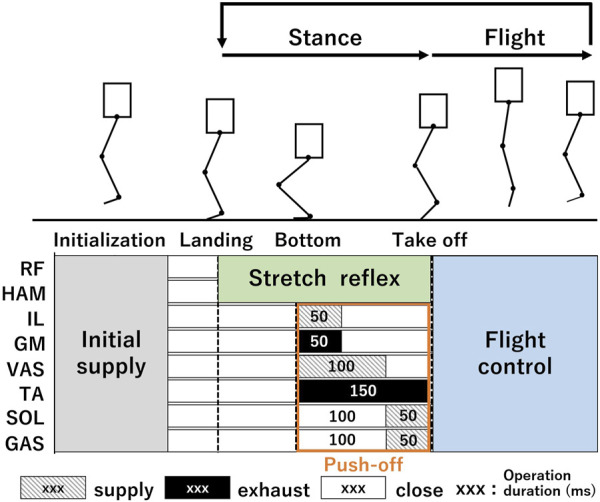
Valve operation commands for each muscle throughout a hopping cycle. Before hopping starts, all PAMs are initialized to a specific air pressure. The RF and HAM are implemented through the stretch reflex system from landing to takeoff (balance function). Other muscles (the setup is shown in Figure 6A) help absorb the landing impact with the passive compliance and initiate a push-off action with a consistent air supply pattern when the robot reaches its bottom point (stance function). After takeoff, all muscles revert to their initial air supply pattern mid-air, preparing for the next landing (flight function). This cycle from landing to takeoff continues.

Complementing the balance function, distinct stance and flight functions have been incorporated for hopping control. Figure 3 shows the respective intervals during which the three functions are activated throughout the hopping sequence. The hopping begins with a free fall, setting all PAMs to their starting position. The position was adjusted such that its knee angle measured 150°, and its feet were parallel to the ground, thereby ensuring a stable landing without compromising the balance (Van Der Krogt et al., 2009). Owing to the inherent compliance of the PAMs, the leg lands flexibly and balance function is immediately activated. The leg, under the influence of gravity, flexes and descends until the reaction force, which is produced by the passive elasticity of the PAMs, counterbalances the gravitational pull—this point, called the bottom point. Consequently, the stance function is activated, leading to a rapid leg extension that produces the vertical momentum required for hopping (push-off). As the leg becomes airborne (takeoff), both the stance and balance functions are deactivated, and the flight function takes over. This cycle, ranging from landing to takeoff, is consistently repeated during a continuous hopping trial.

The stance function encompasses two primary roles: absorbing impact upon landing and executing the push-off action. The passive compliance of the musculoskeletal system contributes to a flexible leg landings, enabling the system to accommodate terrain irregularities (Blickhan, 1989; Geyer et al., 2006; Van Der Krogt et al., 2009). The primary objective of passive compliance is to reduce control efforts and minimize energy consumption during the landing phase. Consequently, muscles aside from the RF and HAM, central to the balance function, remain inactive until the push-off sequence commences. After landing, the robot executes a push-off action from the bottom point, delivering force to the ground to propel itself upward. The bottom point was determined using the methodology from our preceding study, which aimed to optimize the muscle’s utilization of elastic energy (Hosoda et al., 2010). The subsequent determination of air supply and exhaust duration incorporated electromyography (EMG) data recorded during human jumps (Pandy and Zajac, 1991; Petersen et al., 1999), complementing the insights obtained from our earlier study. For this function, muscles excluding RF and HAM (namely, IL, GM, VAS, TA, SOL, and GAS) were employed. A continuous supply and exhaust operation process was conducted, as depicted in Figure 3. Beyond the direct influence of the VAS on the push-off action, the activation timing of the GAS is deemed crucial in achieving optimal hopping height (Babič and Lenarčič, 2006). The GAS is instrumental in transmitting the jump force from the knee joint, through the ankle joint, and finally to the ground (Bobbert and van Ingen Schenau, 1988). The optimal moment for GAS activation was ascertained via a set of preliminary tests, which concluded in a purposeful delay of 100 ms post the inception of the push-off action. To prevent any interference with the GAS’s motion, the SOL activation was coordinated to coincide with that of the GAS.

Flight control is executed to set the appropriate posture of the leg in preparation for the upcoming landing. The landing position of the foot is not actively regulated in this context. The underlying dynamics are fundamentally ballistic, with the landing position determined by the ejection angle resulting from the push-off action. We implemented a straightforward PID control to restore the air pressure within each muscle to its initial state, thus returning the leg to a predetermined posture. The control methodology is predicated based on the following: if the present air pressure exceeds the initial state, the valve is opened to release air. Conversely, if the pressure is low, the valve opens to admit air. The valve is shut when the discrepancy lies within ±5% of the initial state pressure. This flight control approach remains active until the robot achieves the next landing.

### 2.3 Phase transition

Three specific events are discerned by the robot: landing, the bottom point, and takeoff. From previous studies (Hosoda et al., 2010; Raibert, 1986), we see that the event detection utilized the tension within the VAS muscle. An alternative method for detecting these events can be achieved by integrating of switches or a force-sensing resistor (FSR) on the foot’s sole, a strategy that has been extensively investigated (Liu et al., 2018). Nevertheless, our robot’s foot is designed as a simple planar surface coated with anti-slip rubber, devoid of any mechanism to absorb landing shocks directly. Physical switches, in such a setup, are vulnerable to potential damage. Moreover, while force and torque sensors based on strain gauges have been developed (Käslin et al., 2018), these often add significant weight, compromising the robot’s performance. Consequently, we selected a method that detects events using the muscles’ inherent proprioceptive information, particularly tension, which eliminates the need for foot sole sensors. The detection processes are as follows: landing is determined using Eq. 3, the bottom point is ascertained via Eq. 4, and takeoff is discerned through Eq. 5.

$$F_{\text{VAS}}\left(t\right)≧\beta$$ (3)

$$\frac{\partial F_{\text{VAS}}\left(t\right)}{\partial t}=0$$ (4)

$$F_{\text{VAS}}\left(t\right)=0$$ (5)

where *F*_VAS_ is the VAS tension, and *β* is the landing detection threshold. The threshold underwent an immediate update following the reset of the air pressure during the flight phase, being set to the observed tension plus 1 kg. This 1 kg increment acts as the minimal buffer necessary to ensure dependable detection. Upon landing, gravitational force causes the robot’s knee joint to flex, thereby stretching the VAS and amplifying its tension. Such tension surge can be identified using Eq. 3. The VAS tension peak stabilizes as the robot descends to the bottom point, and this stabilization can be pinpointed using Eq. 4. The periodic update of sensor values within each hopping cycle was implemented to address a technical challenge related to landing detection. Interestingly, this process shares a resemblance to the function of *γ* motor neurons in muscle spindles (Brown and Matthews, 1966; Boyd and Gladden, 1985; Guertin et al., 1995). Similar to how muscle spindles use signals from *γ* motor neurons to pretension the intrafusal muscle fibers and automatically adjust sensitivity, our system employs a comparable mechanism for enhanced landing decision techniques.

During the takeoff phase, a push-off motion resulted in the knee’s full extension. Unlike the landing phase, the tension within the VAS dramatically drops to almost nil, as the robot ceases to experience the Ground Reaction Force (GRF). Consequently, the moment when the VAS tension reaches zero is termed the “takeoff timing.”

**FIGURE 4:**
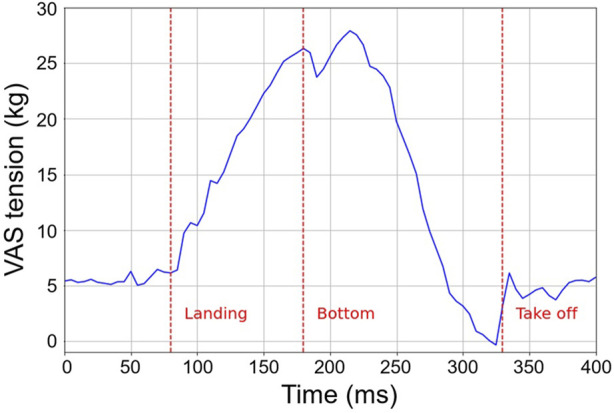
Fluctuations in tension within the VAS throughout the hopping motion. The red markers indicate the moments of the robot’s landing, its bottom point, and takeoff.

Figure 4 presents a typical time-series dataset that captures the tension variations in the VAS, sourced from preliminary trials. The red line signifies the fulfillment of each conditional equation, and the corresponding phase transition takes place at those precise instances. Notably, two tension peaks are discernible around the bottom point. The latter peak, which surfaces around the 360 ms mark, is consequent to the push-off action instigated by the former peak. A subsequent tension of approximately 5 kg, emerging right post takeoff, matches the initial pre-tension applied to the VAS. This indicates the immediate execution of the valve operations associated with the flight function upon takeoff.

## 3 Robot design

### 3.1 Hardware design

**FIGURE 5:**
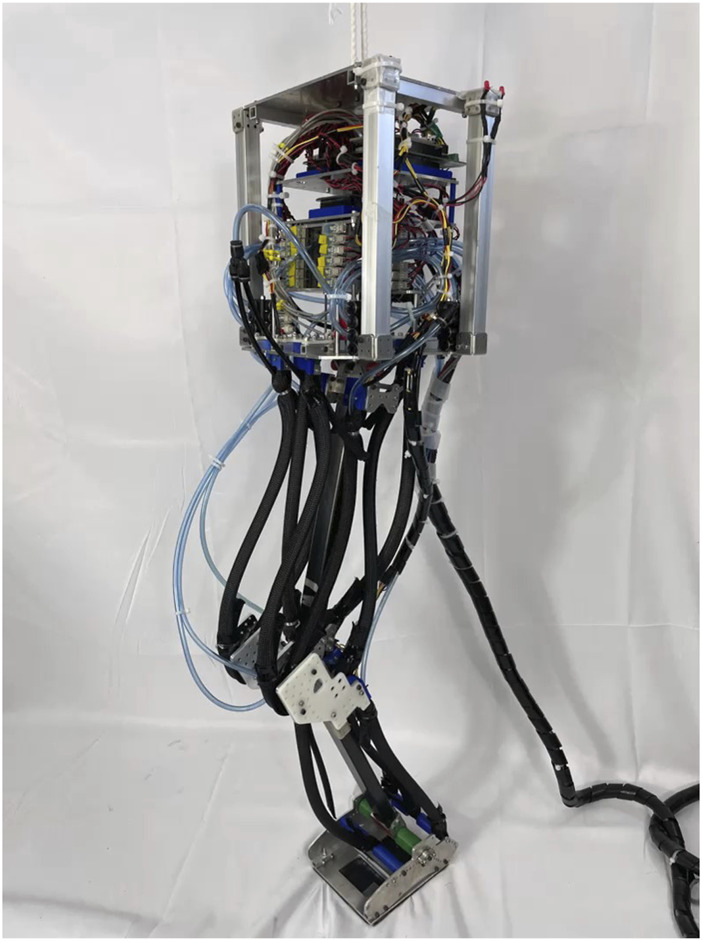
PAM-driven hopping robot.

We developed a PAM-driven hopping robot, as shown in Figure 5. This robot incorporates a trunk and three distinct leg segments: thigh, tibia, and foot, interconnected via three joints: hip, knee, and ankle. The hardware parameters, pivotal to the robot’s design, are shown in Table 1. The PAMs for the biarticular muscles were fabricated from a cutting-edge silicon rubber tube-based PAM. Renowned for the high output-weight ratio (De Volder et al., 2011), PAMs exhibit characteristics qualitatively similar to human muscles, positioning them as compliant linear actuators. By amplifying the internal air pressure, PAMs undergo contraction and produce tension forces. Importantly, the torque at the joints is depends on both the generated tension force and PAM’s moment arm. To guarantee adequate force provision, two PAMs were strategically aligned in a parallel, symmetrical manner for both the antigravity (VAS and SOL) and biarticular (RF and HAM) muscles. Air compression within the PAMs was operated by a solenoid valve, the VQZ132-6M1-C6, produced by SMC. The air pressure’s upper threshold was calibrated to 0.6 MPa.

**TABLE 1:** Hardware parameters.

Parameter	Value [unit]
total mass	6.5 [kg]
trunk length	0.26 [m]
trunk weight	4.0 [kg]
thigh length	0.35 [m]
thigh weight	0.52 [kg]
shank length	0.35 [m]
shank weight	0.52 [kg]
foot length	0.13 [m]
foot weight	0.45 [kg]

**FIGURE 6:**
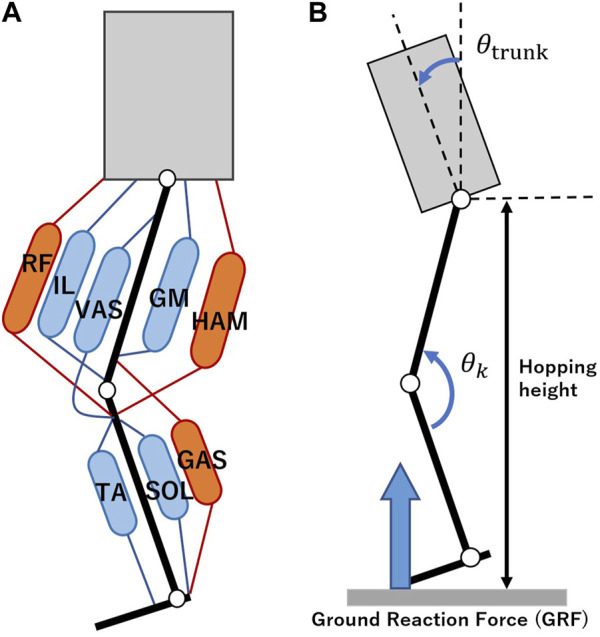
**(A)** Muscle setup. The robot is equipped with eight muscle types, RF: rectus femoris, IL: iliopsoas, VAS: vastus lateralis, GM: gluteus maximus, HAM: hamstrings, TA: tibialis anterior, SOL: soleus, and GAS: gastrocnemius. Biarticular and monoarticular muscles are depicted in red and blue, respectively. RF and HAM are positioned such that their moment arms maintain a 2:1 ratio at the hip and knee joints, particularly 90 mm and 45 mm for RF, and 60 mm and 30 mm for HAM. **(B)** Evaluation metrics. Definitions for the trunk pitch angle, knee angle, hopping height, and GRF are provided.

The muscle setup is shown in Figure 6, which shows 12 PAMs spanning eight categories, all considered integral to the sagittal plane motion. The biarticular muscles, RF, HAM, and GAS, denote the rectus femoris, hamstring, and gastrocnemius respectively. The monoarticular muscles, specifically IL, GM, VAS, TA, and SOL, correspond to the iliopsoas, gluteus maximus, vastus lateralis, tibialis anterior, and soleus, respectively. For an in-depth exploration of each muscle’s role, one can refer to our previous studies for further details (Hosoda et al., 2010). Two distinct sensor types were integrated with the PAMs: tension sensors and air pressure sensors. We developed tension sensors and serially connected to the PAMs, which facilitated the retrieval of tension metrics for five of the eight muscle categories, namely, RF, HAM, GAS, VAS, and SOL. For all PAMs, air pressure was measured using the SMC PSE530 sensors.

### 3.2 Control system configuration

**FIGURE 7:**
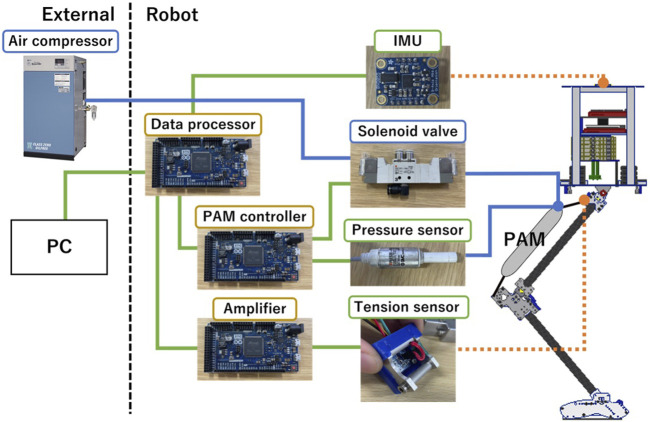
System hardware configuration. The robot is equipped with sensors, actuators, and control boards, while the air compressor and main computer are external installations.

Figure 7 depicts the architecture of the control system. It combines three Arduino Due boards, an array of sensors, actuators, and a primary PC controller. The triad of Arduino boards has distinct roles: interpreting tension sensor signals, actuating the solenoid valves, and processing the data. Furthermore, we designed a bespoke circuit board compatible with Arduino Due to interpret signals from tension sensors. These sensors collected data at a sampling rate of 250 Hz, which was subsequently relayed to the remaining two boards via CAN protocol. The MCP2551 functioned as the CAN transceiver, while the solenoid valve driving board managed valve operations at a frequency of 1 kHz. A 9-axis IMU (Adafruit BNO055) was mounted in the center of robot’s trunk, allowing for the capture of posture metrics, trunk pitch angle, and angular velocity. The data collated by the data processing Arduino was transmitted to a PC via serial communication at a rate of 200 Hz, identified as the optimal frequency for stable, uninterrupted data transfer to the PC.

## 4 Results

To verify the effectiveness of the proposed stretch reflex system, we conducted three experimental paradigms: a single hop experiment, a continuous hopping trial, and a series of hopping experiment with various control gains. In the first and second experiments, the performance of hopping was contrasted between two scenarios: one where the biarticular thigh muscles were incited by the stretch reflex, and the other where they maintained a consistent air pressure, termed passive control. Passive biarticular muscles are designed to transfer force between multiple joints owing to their inherent compliance. This passive mode is an emulation of outcomes from our previous studies (Hosoda et al., 2008; Hosoda et al., 2010). In the first experiment, the trunk pitch angle and angular velocity were observed as the evaluation metrics for the postural stability. Additionally, vertical GRF was observed during the hopping action and compared with human data (Wannop et al., 2012) to ensure congruent environmental interactions. In the second experiment, we investigated whether the proposed method increased the number of continuous hopping by enhancing the postural balance. In the third experiment, we examined the trunk pitch angle through hopping trials with nine different sets of control gains. Within this context, the gains chosen for the continuous hopping trials were adjusted by factors of 0.8 and 1.2 to analyze their influence on postural stability. At the commencement of each experiment, the robot was securely held and subsequently released from a height of 750 mm above the terrestrial surface, with the release point being the hip joint. The IMU attached on top of the robot’s trunk facilitated the observation of the trunk pitch angle and angular velocity. The GRF was measured with a force plate (model TF-3040, manufactured by TEC Gihan Co., Ltd.). The evaluation of the hopping height and knee joint angle was realized through video analysis of markers attached to the robot. For reference, and unless specified otherwise, the control parameters employed throughout these experimental phases are systematically cataloged in Table 2.

**TABLE 2:** List of control parameters used in the experiments.

Parameter	Definition	Value [unit]
*P*_0,IL_	Initial air pressure of IL	0.23 [MPa]
*P*_0,GM_	Initial air pressure of GM	0.16 [MPa]
*P*_0,VAS_	Initial air pressure of VAS	0.20 [MPa]
*P*_0,RF_	Initial air pressure of RF	0.10 [MPa]
*P*_0,HAM_	Initial air pressure of HAM	0.095 [MPa]
*P*_0,TA_	Initial air pressure of TA	0.30 [MPa]
*P*_0,SOL_	Initial air pressure of SOL	0.13 [MPa]
*P*_0,GAS_	Initial air pressure of GAS	0.075 [MPa]
*k*_RF_	Control gain of RF	0.17
*k*_HAM_	Control gain of HAM	0.30
*T*_RF_	Reference duration of RF actuation	5.0 [ms]
*T*_HAM_	Reference duration of HAM actuation	5.0 [ms]
*α*	Threshold of muscle stimulation	1.0

### 4.1 Single hop experiment

**FIGURE 8:**
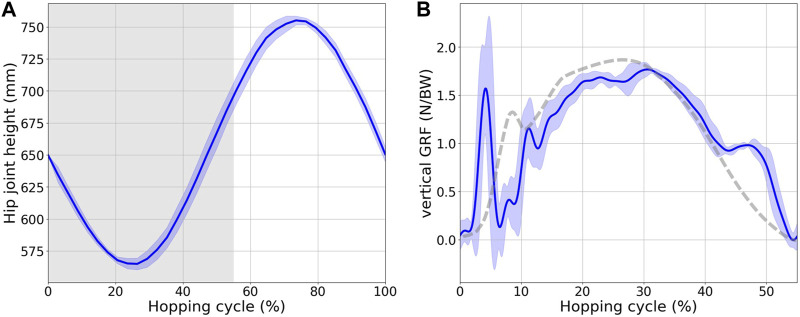
**(A)** Hip joint height during hopping. The x-axis represents one hopping cycle, starting when the robot lands. The gray area indicates the stance phase. **(B)** Vertical GRF over the stance phase of one hopping cycle. The solid blue line shows the GRF observed in the robot hopping experiments, and the dotted gray line is the corresponding human data (Wannop et al., 2012).

The efficacy of the stretch reflex was meticulously assessed via single-hop test trials, with data aggregated across 10 distinct trials. At the onset, an exploratory parameter search was undertaken during the pilot experiment and *k*_RF_ and *k*_HAM_ settled at values of 0.17 and 0.30, respectively. The outcomes of the video analysis, which described the vertical trajectory of the hopping height throughout hopping, are shown in Figure 8A. Both the average values and their respective ±1 standard deviations are marked. The horizontal axis covers from the moment of ground contact upon landing to the subsequent point of contact after a completed hop. The phase during which the robot remains stationed on the ground, termed the stance phase, consistently spanned approximately 55% of the entire hopping cycle, which is delineated by a grey backdrop. The robot initiated its motion with a free-fall from a height of 750 mm above the terrestrial surface and demonstrated the capacity to recoil to this initial height post hop. The vertical GRF is graphically elucidated in Figure 8B. A 50 Hz low-pass filter was employed to alleviate noise interference. The GRF pattern revealed dual peaks, corresponding to the landing and push-off stages. This pattern bears a striking resemblance to the GRF dynamics observed in human hopping (Wannop et al., 2012).

**FIGURE 9:**
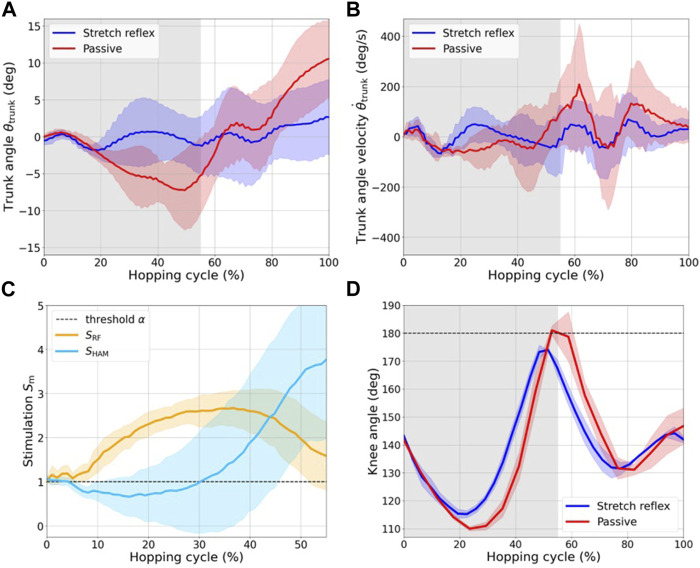
**(A)** Variation in trunk pitch angle, denoted as *θ*_*trunk*_, during hopping. It is essential to note that this experiment involves a single hop, despite the apparent discontinuity at the beginning and end of the cycle presented here. **(B)** Trunk angular velocity, denoted as *θ*_*trunk*_^˙^. The gray area indicates the stance phase. **(C)** Muscle stimulation *S*_m_(*t*) in RF and HAM during the stance phase. The black dashed line marks the threshold *α* for muscle stimulation. **(D)** Comparison of the knee joint angle trajectory over one hopping cycle. The activation of the HAM, facilitated by the stretch reflex, effectively prevented hyperextension of the knee joint beyond 180°.

Comparative analysis of the trunk pitch angle and angular velocity, both with and without the stretch reflex, is shown in Figures 9A, B. The angle was deemed positive when the robot exhibited a forward lean relative to the vertical axis. The integrated stretch reflex effectively reduced the trunk angle perturbation within the confines of ±5°. In stark contrast, the passive control configuration exhibited a backward deviation surpassing 10° from the vertical alignment. This backward lean, observed during the stance phase under passive control, seemingly underwent a compensatory forward shift upon the push-off, resulting in an exaggerated forward lean post takeoff. The stretch reflex demonstrated efficacy beyond the initial 20% of the hopping cycle, which mitigated substantial perturbations by reducing the backward lean during the stance phase. Consequently, the methodology also resulted in a marked reduction in angular velocity.

Figure 9C shows the magnitude of the muscle stimulation exerted on the RF and HAM muscles. The black dashed line indicates the threshold *α* of muscle stimulation, which indicates that the muscles receive an air supply when the stimulation surpasses this specified threshold. The horizontal axis is solely representative of the stance phase. The results show that during the early stance phase, the RF receives an air supply in response to an excitatory muscle stimulation. Further, the RF plays an instrumental role in facilitating knee extension, which aids the push-off action. This role is corroborated by the observation that the minimal knee joint angle, as seen in Figure 9D, was notably larger when the stretch reflex was employed. The RF was always supplied air throughout the stance phase as a result of modifying the control gain during the pilot study, aiming at counteracting the backward lean of the trunk caused by the passive control. This particular challenge can be adeptly addressed by calibrating the initial air supply for each muscle. Throughout the early to mid-stance phase, stimulation of the HAM muscle decreased, owing to the flexing of the knee and a consequent reduction in the HAM tension. In the late stance phase, while the stimulation to the RF muscle decreased, the stimulation of the HAM increased owing to the knee extension facilitated by the push-off action, coupled with the forward lean of the trunk due to the RF contraction. Notably, the HAM contraction appears pivotal in offsetting the forward lean of the trunk and forestalling excessive knee extension, which might arise due to the torque generated by the VAS, as demonstrated in Figure 9D.

### 4.2 Continuous hopping trial

**FIGURE 10:**
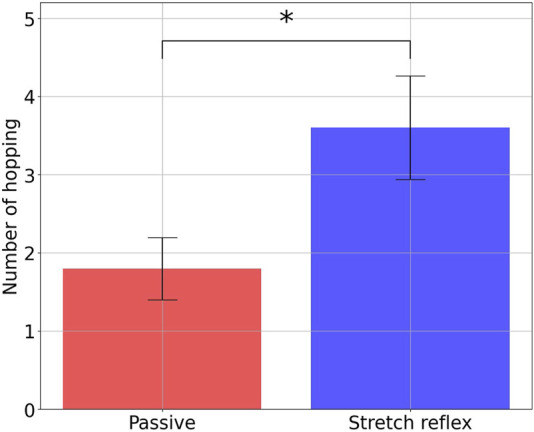
Comparison of the number of continuous hops executed with and without the stretch reflex. There is a significant difference between the conditions (*p < 0.01 after a two-tailed unpaired *t*-test).

**FIGURE 11:**
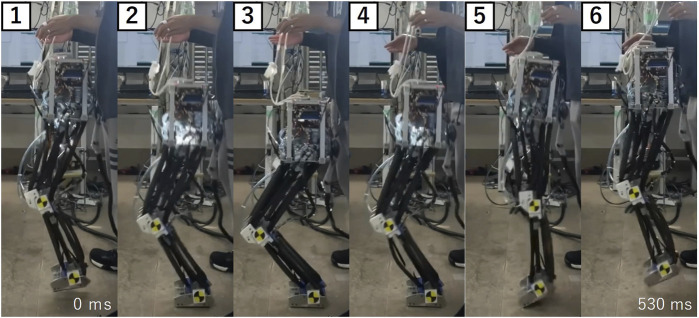
Snapshot from the continuous hopping experiment, detailing one complete hopping cycle. The highlighted cycle spanned 530 ms and the video capture rate is 30 fps.

We compared the number of continuous hops produced under stretch reflex control and passive control to investigate the effectiveness of the trunk angle stabilization using the stretch reflex in genuinely improving hopping efficacy. The findings are shown in Figure 10. The bar graph’s pinnacle represents the average calculated over 10 trials, while the error bar shows the scope of ±1 standard deviation. In the passive control scheme, the mean number of hopping attempts stood at 1.8. In stark contrast, the approach presented here recorded an average of 3.6 hops, effectively doubling the performance of the passive control, thereby underlining a marked enhancement in trunk posture stability. Remarkably, the stretch reflex control realized a zenith of five successive hops, establishing a record for PAM-driven robots, as per our knowledge. Figure 11 provides a snapshot of the robot in mid-hop. Preceding a failed hopping attempt, the trunk’s angular orientation deviated from the recurrent cyclical pattern, culminating in the robot landing with a trunk lean nearing 10°.

### 4.3 Postural stability through variations in control gain configurations

**FIGURE 12:**
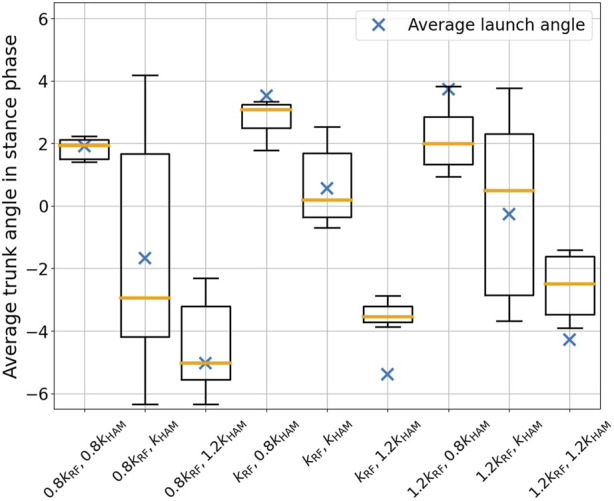
Results of variation in the trunk pitch angle during the stance phase, analyzed in relation to the reflex intensity parameters. The blue “X” indicators depict the mean takeoff angles and the orange line is the median. As the RF gain increased relative to the HAM, the trunk angle tended to lean forward. Conversely, when the gain was decreased, the trunk angle tended to lean backward.

The stretch reflex permits the modulation of the stimulation magnitude derived from muscle tension by adjusting a specific set of control gains, denoted as *k*_RF_, *k*_HAM_. Utilizing the gains *k*_RF_ = 0.17 and *k*_HAM_ = 0.3 from the previously described experiments as benchmarks, we performed nine distinct experiments, each entailing five trials, with gain combinations adjusted by 20% at rates of 80%, 100%, and 120%. These gains predominantly influence the stance phase. The time-series data of the trunk angle during each trial’s stance phase were averaged, and the outcomes were delineated as box-and-whisker diagrams in Figure 12. The blue “X” indicators depict the mean takeoff angles, which typically exhibited greater lean compared to the average stance phase angle. An escalation in the RF gain relative to the HAM resulted in a propensity for the trunk angle to lean forward. Conversely, a reduction in the gain prompted the trunk angle to lean backward. An increase in the HAM gain steered the trunk in a backward orientation regardless of the RF value. Minimal variance was discerned between the cases of 1.2 and 1.0 *k*_RF_. This constancy can be attributed to the consistent air supply to the RF at 1.2 and 1.0, as seen in Figure 9C; however there was no substantial change in the valve operation.

## 5 Discussion

### 5.1 Biarticular thigh muscles and postural stability during robot’s hopping

The results from the single-hop tests show that the stretch reflex applied to the biarticular thigh muscles effectively reduced perturbations in the trunk’s pitch angle. While passive control typically resulted in a backward lean of nearly 10° during the stance phase, the stretch reflex results in a lean under 5°, thereby enhancing the postural stability, which in turn empowered the robot to execute a maximum of five continuous hops. In contrast to conventional motor-driven robots, this particular robot eschews the utilization of posture data from IMUs or encoders for control purposes. According to standard control theory, the hip’s monoarticular muscles, IL and GM, are ideal for stabilizing the trunk angle. In theory, postural stabilization can be achieved using these muscles, provided the leg’s entire trajectory is computed from the individual joint angle information and appropriate hip torque is ascertained through inverse kinematics. However, the considerable output delay inherent in PAMs compared to motors makes accurate hip torque tracking by PAMs highly challenging. Humans overcome this complication by introducing redundant degrees of freedom via biarticular muscles. The rectus femoris (RF) functions as a hip flexor and knee extensor, while its antagonist, the hamstring (HAM), assumes inverse roles. Their antithetical roles at each joint enable them to influence the trunk pitch angle, which is independent of the leg’s state. Upon the leg’s landing, the hip joint extends and the knee flexes, with the trunk descending. Though GM, a monoarticular muscle, extends in tandem with the leg’s movement, the HAM’s motion is characterized by simultaneous hip extension and knee flexion. Consequently, the overall state of the HAM, whether it is in an extended or relaxed form, is determined by each joint’s moment arm. Prior studies show that when the biarticular muscles’ moment arms to the hip and knee are in a 2:1 ratio and both the thigh and lower leg possess equal lengths, the movement of individual joints exerts no impact on them (Lakatos et al., 2014; Hosoda et al., 2017). By designing the robot adhere to these principles, only the trunk angle is influenced by the contraction of the biarticular thigh muscles. It is imperative to highlight that this scenario holds true only when the robot bears significant weight, ensuring substantial friction between the ground and legs. In the absence of this weight, muscle contraction primarily induces significant movement in the entire leg, rather than the trunk alone. The employment of biarticular thigh muscles for postural stabilization has also been corroborated as an inherent mechanism in human control modalities, as evidenced by human EMG measurements (Schumacher et al., 2019). We reproduced this mechanism by implementing stretch reflexes into a robot. Despite the complex dynamics associated with the posture control of a hopping robot, the introduced stretch reflex system reframes this issue into a form of PD control parameter search challenge. This reconceptualization renders the problem notably more straightforward than solving the inverse hopping kinematics.

The trunk angle dynamics are contingent upon the physical state of the robot. The intrinsic hopping dynamics can be inferred from the trunk angle under passive balance control. For our robot, the stance phase exhibited a backward leaning dynamic, shifting to a forward leaning dynamic post takeoff. Suitable feedback coefficients to stabilize this dynamic were identified in a preliminary experiment, resulting in values of 0.17 and 0.3 for RF and HAM, respectively. The parameter set are specifically designed for our robot and are relevant only under the conditions of the conducted experiments. For example, merely altering the natural length of a singular PAM by a few centimeters and reattaching it can transform the intrinsic hopping motion dynamics, thereby necessitating a retuning of the parameters. Furthermore, the parameter set would need to be retuned. However, the stretch reflex mechanism is typically effective in mitigating excessive trunk perturbations in hopping dynamics and can be adapted to robots of varying dynamics by simply recalibrating the parameters.

### 5.2 Toward the progress of PAM-driven legged robots

The stretch reflex serves as a rapid and straightforward feedback mechanism. This study represents a successful endeavor to incorporate a feedback system into PAM to improve the controllability of PAM robots. While feedback systems have been primarily integrated into robotic arms with several instances of precise trajectory control, reported locomotion presents the challenge of substantial interaction with external environments, leading to difficulty in estimating the dynamics produced by the PAM. Historically, establishing an effective feedback system for locomotion mechanisms has remained elusive, thereby hindering significant advancements in the domain, especially since 2010 when research predominantly focused on the body’s physical attributes. To overcome this situation, this study employed a balance function, rooted in the stretch reflex of the biarticular thigh muscles, RF and HAM, on a PAM-driven legged robot. A pivotal challenge in devising a feedback system for a PAM-driven legged robot is the myriad of factors that can compromise the correlation between the input PAM air pressure and the robot’s trajectory output in complex systems like locomotion. Traditional model-based controls, which are staples in conventional robot control, fall short in such scenarios. Given the dynamics of PAMs that closely resemble human muscles, it is logical to develop PAM control systems with cues from human neuromuscular control.

While various theories have been proposed about the principles of human locomotion control, an impeccable solution remains elusive. Unlike conventional robot control, human locomotion does not demand highly precise trajectory planning. The inherent dynamics of the body allow for a certain degree of control error. Concurrently, the neural system ought to harness its experiential knowledge to adjust muscle output and facilitate stable movements. To incorporate this comprehensive locomotion principle into PAM control methodology, we focused on the spinal reflex mechanism. These reflexes coordinate the stimulation to motor neuron by modulating sensory signals via interneurons, which play a pivotal role in muscle activation. The amplitude of the muscle activation instigated by the reflex is tethered to the sensory signal’s magnitude. This symbiotic relationship between the sensory signal and muscle activation undergoes adjustments during routine activities. Drawing inspiration from the reflex mechanism, coupled with the recently introduced notion of positive force feedback (Geyer et al., 2003; Sharbafi and Seyfarth, 2015), we designed a system that seamlessly translates muscle tension into the duration of air supply necessary for PAM activation, as depicted in Figure 2. The effectiveness of the stretch reflex system for PAM-driven robots demonstrated in this study paves the way for an array of future applications.

### 5.3 Summary and potential future directions

Previous studies have proposed the emulation of human morphological attributes using PAMs as a viable design strategy for legged robots. However, the complex dynamics of PAMs present a formidable challenge in constructing a suitable locomotion model. Furthermore, PAMs have typically been deemed unsuitable for feedback-system-requisite tasks, including postural stabilization.

To demonstrate the potential applicability of feedback systems to PAMs, this study introduced a balance function inspired by the human stretch reflex mechanism to facilitate postural stabilization during the hopping cycles of a PAM-driven robot. Our array of hopping experiments yielded several observations:

1. Implementing the stretch reflex system on the biarticular thigh muscles (RF and HAM) facilitates muscle activation in response to perturbations in the trunk angle caused by pitch leaning.
2. The consequent muscle contraction successfully mitigates the perturbation, achieving trunk stabilization throughout the hopping cycle.
3. The direct consequence of utilizing the stretch reflex system is an augmented ability to execute continuous hops.
4. The reflex response intensity can be modulated by altering the gain associated with the stretch reflex in the biarticular muscle pairs.

Despite its successes, the proposed system does not fully replicate the intricate nature of the stretch reflex mechanism owing to technological constraints. The reflex is predominantly initiated due to fluctuations in the muscle length, while our configuration utilized muscle tension. In human physiology, muscle tension is monitored through Golgi tendon organs to mediate excessive muscle strain. This proposes a rationale for a system devised to receive length as the excitatory signal and tension as the inhibitory one for individual muscles. Realizing a more precise model of the stretch reflex in PAM-driven robots could potentially forge a tool conducive for exploring the functionalities of human locomotion through a constructivist lens. Although existing anatomical investigations and human experiments have delineated the neural pathways steering locomotion, the overarching impact remains elusive due to the inseparable intertwining of other neural and cognitive processes in living organisms (Pfeifer and Bongard, 2006; Spröwitz et al., 2013; Ijspeert, 2014). For physical simulations, body dynamics including the landing impact are still very complex, making it difficult to construct a proper model within a computational environment. Therefore, it has not been a sufficient tool for exploring this issue (Ijspeert, 2014). In the future, building upon this foundational research may position PAM-driven robot experiments as a robust method for decoding complex human locomotion.

## Data Availability

The raw data supporting the conclusion of this article will be made available by the authors, without undue reservation.
